# Efficacy of a Multi-Component m-Health Diet, Physical Activity, and Sleep Intervention on Dietary Intake in Adults with Overweight and Obesity: A Randomised Controlled Trial

**DOI:** 10.3390/nu13072468

**Published:** 2021-07-19

**Authors:** Sasha Fenton, Tracy L. Burrows, Clare E. Collins, Anna T. Rayward, Beatrice Murawski, Mitch J. Duncan

**Affiliations:** 1 Priority Research Centre for Physical Activity and Nutrition, The University of Newcastle, University Drive, Callaghan, NSW 2308, Australia; sasha.fenton@uon.edu.au (S.F.); tracy.burrows@newcastle.edu.au (T.L.B.); clare.collins@newcastle.edu.au (C.E.C.); anna.rayward@newcastle.edu.au (A.T.R.); beatrice.murawski@uon.edu.au (B.M.); 2 School of Medicine and Public Health, College of Health, Medicine and Wellbeing, The University of Newcastle, University Drive, Callaghan, NSW 2308, Australia; 3 School of Health Sciences, College of Health, Medicine and Wellbeing, The University of Newcastle, University Drive, Callaghan, NSW 2308, Australia; 4 School of Education, College of Human and Social Futures, The University of Newcastle, University Drive, Callaghan, NSW 2308, Australia

**Keywords:** diet, nutrition, physical activity, sleep, overweight, obesity, weight loss, RCT, m-health

## Abstract

This three-arm randomised controlled trial evaluated whether (1) a multi-component weight loss intervention targeting diet, physical activity (PA), and sleep was effective at improving dietary intake over six months and 12 months, compared with a control, and (2) the enhanced diet, PA, and sleep intervention was more effective at improving dietary intake than the traditional diet and PA intervention. A total of 116 adults (70% female, 44.5 years, BMI 31.7 kg/m^2^) were randomised to either traditional diet and PA intervention; enhanced diet, PA, and sleep intervention; or wait-list control. To examine between-group differences, intervention groups were pooled and compared with the control. Then, the two intervention groups were compared. At six months, the pooled intervention group consumed 1011 fewer kilojoules/day (95% CI −1922, −101), less sodium (−313.2 mg/day; 95% CI −591.3, −35.0), and higher %EI from fruit (+2.1%EI; 95% CI 0.1, 4.1) than the controls. There were no differences in intake between the enhanced and traditional groups at six months. At 12 months, the pooled intervention and control groups reported no significant differences. However, compared to the traditional group, the enhanced reported higher %EI from nutrient-dense foods (+7.4%EI; 95% CI 1.3, 13.5) and protein (+2.4%EI; 95% CI 0.1, 4.6), and reduced %EI from fried/takeaway foods (−3.6%EI; 95% CI −6.5, −0.7), baked sweet products (−2.0%EI; 95% CI −3.6, −0.4), and packaged snacks (−1.1%EI; 95% CI −2.2, −0.3). This weight loss intervention reduced total energy and sodium intakes as well as increased fruit intake in adults at six months. The enhanced intervention group reported improved dietary intake relative to the traditional group at 12 months.

## 1. Introduction

In 2015, 39% of the global population was overweight or obese [1]. Excess adiposity increases the risk of chronic diseases, such as type two diabetes and cardiovascular disease, decreases life expectancy, and has negative personal, social, and economic consequences [2]. Contributing to the prevalence of overweight and obesity is poor diet quality [3], which is characterised by high consumption of energy-dense, nutrient-poor foods and drinks containing saturated fat and/or added salt and sugar, and alcohol; with inadequate consumption of fruits, vegetables, and whole grains [4,5]. Given the association between poor diet quality and weight gain [3], aligning dietary patterns with national guidelines is a recommended strategy for treatment of overweight and obesity, along with increasing physical activity [6]. Weight loss interventions most commonly target reduced total fat and increased fruit and vegetable intakes to create an energy deficit [7,8,9]. However, weight loss studies rarely report participants’ dietary changes in addition to weight loss outcomes [10,11]. Of those studies that have reported dietary outcomes, change in total energy intake or macronutrient intake was most commonly reported, while change in overall diet quality or foods was rarely reported [12]. While changes energy and macronutrient intakes are important, identifying changes in the consumption of different types of healthy/unhealthy foods provides valuable information about food patterns that are amenable to change in the context of overweight and obesity. This information could be used to inform future weight loss interventions and may assist in improving effectiveness.

Recently, sleep has been recognised as another health behaviour that is potentially modifiable for weight management [13,14]. Short sleep duration has been linked to poor dietary behaviours with several experimental studies reporting increased energy intake, higher number of meals and snacks consumed, and increased night-time eating in response to short sleep duration [14,15,16,17]. The physiological mechanisms that drive increased food intake and energy intake following short sleep are not well understood, but short sleep appears to increase activity in the brain’s hedonic (reward) system. Evidence from experimental studies suggests that during periods of restricted sleep duration, the presentation of food stimuli provokes greater activity patterns in brains areas associated with pleasure and reward, compared with viewing the same food stimuli following normal sleep [18,19,20,21,22]. Another proposed mechanism is through the regulation of appetitive hormones. Several experimental studies have reported elevated concentration of the hunger-stimulating hormone ghrelin and reduced concentration of the satiety hormone leptin in response to short sleep duration [23,24,25]. In terms of the increase in energy intake that can occur when sleep is insufficient, meta-analyses have reported an increase of 1059–1612 kilojoules (kJ) (253–385 kcal) per day in adults whose sleep was restricted to ≤5.5 h per night, compared with controls [18,26,27]. As such, increasing sleep duration among short-sleepers may contribute to reducing energy intake and improving energy balance [28] for enhanced weight loss.

Few randomised controlled trials (RCTs) have measured the effect of a dedicated sleep intervention on dietary intake [29,30,31,32]. One RCT (*n* = 42) measured the effect of extended sleep duration on aspects of dietary intake including energy, macronutrient, sugar, fibre, and caffeine intake and diet quality over four weeks in adults with normal weight [29]. The study reported that extended sleep duration reduced free-sugar intake and increased adherence to dietary guidelines [29]. Another RCT (*n* = 22) that measured the effect of six weeks of extended sleep duration compared with control on energy, macronutrient, and sodium intake for eight days reported no significant change in dietary outcomes [30]. However, preliminary data from an RCT (*n* = 60) in adults with overweight and obesity has shown that two weeks of sleep extension (≈1.2 h per night) resulted in significantly lower energy intake (−207 kcal per day), compared with control [31]. There is limited evidence investigating dietary outcomes in response to a sleep intervention over longer periods.

The primary objective of the current study was to evaluate whether a multi-component m-Health weight loss intervention in adults with overweight and obesity that targeted dietary, physical activity, and sleep behaviours was effective at improving dietary intake over six months, and longer-term at 12 months, compared to a wait-list control. The secondary objective was to evaluate whether the intervention targeting dietary, physical activity, and sleep behaviours (enhanced) was more effective at improving dietary intake than the dietary and physical activity intervention (traditional). The hypotheses were that the enhanced and the traditional interventions would achieve greater improvements in dietary intake than the wait-list control at six and 12 months, and that the enhanced intervention would achieve greater improvements in dietary intake than the traditional intervention.

## 2. Materials and Methods

### 2.1. Trial Design

This is a secondary analysis of the Move, Eat & Sleep study, which has been described in detail elsewhere [33]. Move, Eat & Sleep was a 3-arm parallel-group RCT with a six-month intervention period (primary endpoint) and follow-up at 12 months. Participants were recruited May–September 2017 from Newcastle, NSW, Australia, primarily by media stories, social media advertising, and participant registries. Sample size calculations were based on the primary outcome measure, weight (kilograms), as described elsewhere [34]. A minimum of 114 participants was required to provide adequate power, and a total of 116 were randomised. Participants were stratified by baseline body mass index (BMI) (25.0–29.9, 30.0–40.0 kg/m^2^) and randomly allocated (1:1 ratio) by the project manager to one of three groups using secure, web-based, permuted block randomisation that was generated by an independent statistician. Ethical approval was granted by the Human Research Ethics Committee of the University of Newcastle (H-2017–0039), and the trial was prospectively registered with the Australian New Zealand Clinical Trials Registry (ACTRN12617000735358; U1111-1219-2050). Informed written consent was obtained from all individual participants included in the study. The reporting of this study adheres to the Consolidated Standards of Reporting Trials (CONSORT) guidelines [35].

### 2.2. Participants

Individuals were eligible for participation if they reported being aged 18–65 years, a BMI of 25.0–40.0 kg/m^2^ (overweight/obesity classification), had access to an iOS/Android device with Internet access, and had the ability to attend four assessments over 12 months. Individuals were ineligible if they were using a tracking device for physical activity and/or sleep, were pregnant, reported the presence of a sleep disorder diagnosed by a medical practitioner, were taking medication related to sleep or weight management, had a condition that precludes participation in physical activity and/or modification of diet and/or sleep, had lost ≥4.5 kg of body weight in the last three months, intended to participate in another weight loss program, had previous weight loss surgery, or were employed as a shift-worker (on a rotating roster).

### 2.3. Intervention

The intervention components have been described in detail previously [34]. Briefly, the Move, Eat & Sleep study was a multi-component m-Health behaviour-change weight loss intervention. The traditional intervention group targeted change in dietary and physical activity behaviours, while the enhanced intervention group targeted change in dietary behaviours, physical activity, and sleep health. The wait-list control group received access to the intervention after the 12 month follow-up. Participants received intervention content specific to their group allocation and self-directed under free-living conditions. The intervention content was guided by specific behaviour change techniques (e.g., goal-setting, self-monitoring) to operationalise constructs from the self-regulatory and social cognitive theories [36,37,38].

The dietary intervention was delivered via intervention materials and one face-to-face dietary session with a dietitian, where participants were given personalised dietary advice based on assessment of their current dietary intake, as measured by the Australian Eating Survey^®^ (FFQ) and personalised nutrition report (Australian Eating Survey^®^ Version 2, The University of Newcastle, Callaghan, NSW, Australia) [39,40]. They received information about the Australian Dietary Guidelines and the Australian Guide to Healthy Eating, which promote increased intake of nutrient-dense core food groups and reduced intake of energy-dense, nutrient-poor discretionary foods and drinks [41]. Resources for planning healthy meals, controlling portion sizes, and interpreting food labels were also provided. Participants were given a personalised daily energy intake target of 2000 kJ less than their estimated energy requirement to create an energy deficit, as this is the amount required to achieve ≈0.5 kg weight loss per week [42]. Intervention group participants were instructed to set dietary behaviour goals and self-monitor their behaviours using the Balanced app, which contains 10 daily food goals: (1) eat two serves of fruit, (2) eat five serves of vegetables, (3) choose whole-grains, (4) choose low-fat dairy, (5) choose lean meats/alternatives, (6) have a soft drink/energy drink-free day, (7) have an alcohol-free day, (8) choose healthy snack options, (9) have a fast-food-free day, (10) drink plenty of water. Participants were instructed to choose the number of food goals they would aim to achieve daily. This approach has been used previously to improve indicators of diet quality [43]. Participants were instructed to self-monitor their daily energy intake against their recommended energy intake goal using the ControlMyWeight^TM^ app by CalorieKing four days per week (CalorieKing Wellness Solutions Inc., La Mesa, CA, USA). Participants were also recommended to self-monitor and receive feedback about their diet quality by completing the free online Healthy Eating Quiz™ (version 3, The University of Newcastle, Callaghan, NSW, Australia) [44] monthly throughout the intervention period.

The physical activity intervention component promoted increasing daily steps, moderate-to-vigorous intensity physical activity (MVPA), and resistance training (RT) activity to align with physical activity and sedentary behaviour guidelines [45]. Participants were asked to set physical activity goals and self-monitor daily step counts, minutes of physical activity, and number of RT sessions using the Balanced app and a provided Fitbit Alta activity tracker (Fitbit Inc, San Francisco, CA, USA).

The sleep intervention component delivered to the enhanced intervention group provided information about sleep duration and quality recommendations, the importance of sleep health, and daily sleep hygiene practices for participants to implement to promote healthy sleep. Information on stress management techniques (i.e., progressive muscle relaxation, deep breathing, mindfulness), and guidance on cognitive (e.g., self-efficacy, outcome expectations, goal-setting, action planning, self-monitoring, feedback) and behavioural self-regulation strategies (e.g., sleep hygiene practices such as limited bed and wake time variability, limiting caffeine consumption) to promote sleep health was also provided [34,46,47]. Enhanced group participants were asked to set and self-monitor sleep goals for sleep time, wake time, and sleep hygiene practices to help them achieve adequate sleep duration, consistent sleep timing, and improved sleep quality. To promote compliance and reinforce the intervention components, participants received personalised weekly feedback about their progress in relation to their goals.

### 2.4. Measures

All outcome measures were completed at baseline, six months, and 12 months at The University of Newcastle, Australia by trained assessors using a standardised protocol. Assessors were blinded to participant group allocation at each time point, and participants were blinded to group allocation until the completion of baseline assessments.

#### 2.4.1. Sociodemographics and Anthropometry

Sociodemographic data were collected at baseline using the Qualtrics^XM^ online survey platform (Qualtrics, Provo, UT, USA). Weight and height were measured to the nearest 0.1 kg and 0.1 cm, respectively, on a combined stadiometer and calibrated digital scale (Biospace BSM370 Portable Automatic BMI Stadiometer, Biospace Co, Ltd., Seoul, Korea) and used to calculate BMI (weight (kg)/height (m^2^)). Waist circumference was measured to the nearest 0.5 cm using a measuring tape (Seca 203, Seca Gmph & Co., Hamburg, Germany). Glycated haemoglobin (HbA1c) was measured using a capillary blood sample and analysed using the validated A1C Now+ device (Polymer Technology Systems).

#### 2.4.2. Dietary Intake

Dietary outcomes (energy intake, macronutrient intake (including alcohol), micronutrient intake, nutrient-dense food intake, energy-dense nutrient-poor food intake, diet quality, and caffeine intake) were measured using the Australian Eating Survey (AES), which is a validated 120-item semi-quantitative FFQ that measures the frequency of consumption of foods items and types over the previous three to six months [39,40]. Standard adult portion sizes were derived from the 1995 National Nutrition Survey data or from the product standard serving size. Nutrient intakes were calculated from the most current food composition database of Australian foods, the AusNut 1999 database (All Foods), and AusFoods (Brands) Revision 5. The AES also generates a diet quality score, the Australian Recommended Food Score (ARFS) calculated from a subset of AES questions [39,40]. Diet quality scores are based on core food groups recommended in the Australian Dietary Guidelines [39,48] and calculated by summing points per food item. The score range is from 0 to 73, with higher scores indicative of higher diet quality and categorised as: ‘needs work’ (<33), ‘getting there’ (33–38), ‘excellent’ (39–46), or ’outstanding’ (47+) [44]. Daily caffeine consumption (drinks containing caffeine) was measured using a question adapted from the AUDIT-C alcohol screening test [49], with minor word changes.

#### 2.4.3. Physical Activity and Sleep Health

Weekly minutes of MVPA were measured using The Active Australia Survey (AAS). The AAS has acceptable levels of validity and test–retest reliability [50,51], and it is sensitive to change [52]. Sleep quality was measured using the Pittsburgh Sleep Quality Index (PSQI) [53]. The PSQI is a 19-item survey that measures indicators of sleep health (sleep duration, sleep onset latency, sleep efficiency, sleep disturbances, daytime dysfunction, sleep medication use, and subjective sleep quality) over the previous month. The scores (0–3) for each sleep indicator are summed to provide an overall sleep quality score ranging from 0 to 21, where ‘0′ indicates no sleeping difficulty and ‘21′ indicates severe difficulty. Sleep quality scores >5 are indicative of poor sleep quality [53]. Sleep duration is calculated from PSQI questions about bed and wake times. The PSQI has demonstrated good reliability (Cronbach *α* = 0.83), sensitivity to change, and strong psychometric properties [53,54].

### 2.5. Statistical Methods

Analyses followed intention-to-treat principles using generalised linear mixed models (GLMM). To examine between-group differences, first, the pooled intervention group (traditional and enhanced) was compared to the control group. The groups were combined to maximise statistical power. Then, the two intervention groups were compared to each other. Group differences in key variables at six months were examined using GLMM using an ANCOVA (baseline-adjusted) approach. The models included fixed effects for the baseline value of the outcome, group, and the BMI stratification variable (categorised as 25 to <30 and 30 to 40 kg/m^2^). Group differences in caffeine intake at both time points were examined using logistic regression with adjustment for the baseline value of the outcome. GLMM were used to measure group differences in dietary intake, physical activity, and sleep quality and duration at 12 months. A random intercept for ID accounted for repeated measures. All models used a response distribution and link function as appropriate to the outcome, with an alpha level of 0.05. Sensitivity analyses explored the influence of participants living in the same residence allocated to the same intervention group. Analyses were conducted using Stata version 15 [55]. Effect sizes were calculated using the equation: Cohen’s *d* = (M_1 change score_ − M_2 change score_)/SD _pooled [change scores]._ The magnitude of effects is interpreted using the criteria, small (0.2), medium (0.5), and large (0.8), as defined by Cohen [56].

## 3. Results

### 3.1. Participants

The Move, Eat & Sleep Study enrolled 116 participants: traditional intervention group, *n* = 41; enhanced intervention group, *n* = 39; wait-list control group, n = 36 (Figure S1). The retention rate was 70% at six months with 81 participants completing dietary assessments (traditional, *n* = 32; enhanced, *n* = 28; wait-list control, *n* = 21), and 47% at 12 months (54 participants: traditional, *n* = 23; enhanced, *n* = 14; wait-list control, *n* = 17). The mean age of participants was 44.5 ± 10.4 years (range 19–65 years), 71% were female and 94% identified as Caucasian. Eighty-seven percent of participants were employed, and 78% reported ≥14 years of education. The mean baseline BMI was 31.7 ± 3.9 kg/m^2^ (obese classification). Participants reported participation in 313 min of MVPA per week. The mean PSQI sleep quality score was 7.0, and the mean self-reported sleep duration was 6.8 h (406 min) per night (Table 1).

### 3.2. Dietary Intake

#### 3.2.1. Baseline

Participants (*n* = 116) had mean (±SD) energy intake of 9683 (±3146) kJ per day, with 43.5% of energy from carbohydrate, 33.3% from fat (14.0% from saturated fat), 18.0% from protein, and 5.6% from alcohol. Nutrient-dense foods contributed 58.3% of total energy intake, with grain foods contributing the most energy (16.7%), followed by meats (13.1%), dairy foods (9.0%), vegetables (8.5%), fruits (6.6%), and meat alternatives (4.4%). Participants reported consuming 4.5 serves of vegetables and 1.8 serves of fruit per day. Participants had a mean ARFS (diet quality score) of 35.4 (±9.0) out of a maximum 73 points, which is categorised as ‘getting there’ [44], and scored the highest for variety within the vegetable subscale. Energy-dense nutrient-poor foods contributed 41.7% of total energy intake, predominantly from fried/takeaway foods (9.1% of total energy), confectionery (6.8%), baked sweet products (5.6%), and alcoholic beverages (5.6%). At baseline, approximately half (52%) of participants reported consuming ≤2 drinks containing caffeine per day, while the remaining participants reported higher caffeine consumption. Overall, dietary intake was similar between all groups at baseline (Table 1).

#### 3.2.2. Six Months and 12 Months

Total daily energy intake: At six months, there was a significant mean difference between the pooled intervention group (traditional and enhanced groups) and the control group in energy intake. The pooled intervention groups consumed 1011 fewer kilojoules (242 kcal) per day compared with the control group (95% CI −1922, −101; *p* = 0.029; Cohen’s *d* = 0.55), indicating a medium-sized intervention effect (Table 2). At 12 months, the pooled intervention group maintained a non-significant reduction in total energy intake compared with the control group (−913 kJ; 95% CI −2033, 207; *p* = 0.110; *d* = 0.47) (Table 3).

Macronutrients: No significant differences were observed between the pooled intervention group and the control group at six months for macronutrient distribution (including alcohol) or fibre intake (Table 2). Effect sizes ranged from *d* = 0.02 to 0.29. At 12 months, the enhanced group reported a significantly higher percentage of energy from protein than the traditional group (+2.4 %EI; 95% CI 0.1, 4.6; *p* = 0.040; *d* = 0.74). No significant between-group differences were observed for energy, macronutrient, or micronutrient intake at 12 months (Table 3).

Micronutrients: The pooled intervention groups reported statistically significantly lower sodium intakes per day compared with the controls at six months (−313.2 mg; 95% CI −591.3, −35.0; *p* = 0.027; *d* = 0.56) (Table 2). At 12 months, the reduction in sodium intake was maintained by the pooled intervention group, but it was not significantly different from the controls (−326.2 mg; 95% CI −662.6, 10.2; *p* = 0.057; *d* = 0.56) (Table 3).

Energy intake from healthy core foods and discretionary non-core foods, and diet quality: There were no significant differences between the pooled intervention group and the control group at six months in nutrient-dense food intakes, energy-dense nutrient-poor food intake, or diet quality, with small–medium effect sizes estimated (*d* = 0.03–0.44). At six months, the pooled intervention group reported a significantly higher percentage of energy from fruits than the control group (+2.1 %EI; 95% CI 0.1, 4.1; *p* = 0.040; *d* = 0.53); however, while still higher than at baseline, the difference in fruit intake between groups was not maintained at 12 months (+1.6 %EI; 95% CI −0.3, 3.5; *p* = 0.093; *d* = 0.47). Caffeinated beverage consumption was also not significantly different at six or 12 months. At 12 months, the pooled intervention group reported significantly lower consumption of fried/takeaway foods relative to the control group (−2.4 %EI; 95% CI −4.7, −0.2; *p* = 0.034; *d* = 0.64).

At six months, there were no differences in intake of nutrient-dense foods, energy-dense nutrient-poor foods, and diet quality (Table 2) between the traditional and enhanced groups (*d* = 0.05–0.52). However, at 12 months, significant differences were observed between the traditional and enhanced groups for eight dietary outcomes, with the enhanced group consuming a higher percentage of energy intake from nutrient-dense foods (+7.4 %EI; 95% CI 1.3, 13.5; *p* = 0.017; *d* = 0.81), protein (+2.4 %EI; 95% CI 0.1, 4.6; *p* = 0.040; *d =* 0.74), and lean meats (+4.5 %EI; 95% CI 0.4, 8.5; *p* = 0.029; *d* = 0.76), and lower percentage of energy intake from energy-dense nutrient-poor foods (−7.4 %EI; 95% CI −13.5, −1.3; *p* = 0.017; *d* = 0.81), fried/takeaway foods (−3.6 %EI; 95% CI −6.5, −0.7; *p* = 0.015; *d* = 0.81), baked sweet products (−2.0 %EI; 95% CI −3.6, −0.4; *p* = 0.014; *d* = 0.85), and packaged snacks (−1.1 %EI; 95% CI −2.2, −0.03; *p* = 0.043; *d* = 0.75). The enhanced group also reported significantly lower serves of fruit than the traditional group at 12 months (−0.8 serves; 95% CI −1.5, −0.2; *p* = 0.011; *d* = 0.90) (Table 3).

## 4. Discussion

The primary objective was to evaluate whether a multi-component m-Health weight loss intervention in adults with overweight and obesity that targeted dietary, physical activity, and sleep behaviours was effective at improving dietary intake over six months, and after 12 month follow-up, compared with a wait-list control. Results indicated that the pooled intervention, relative to the control, was effective in the short term (six months) in achieving significantly lower total energy and sodium intakes and increased fruit intake. Whilst the pooled intervention group maintained these improvements at 12 months, the differences between the pooled intervention and the control groups were not significant. The secondary objective was to evaluate whether the intervention targeting dietary, physical activity, and sleep behaviours (enhanced) was more effective at improving dietary intake than the dietary and physical activity intervention only (traditional). The results of this study show that at six months there were no significant differences in dietary intake observed between the intervention groups. However, at 12 months, the enhanced group reported a significantly higher percentage of energy intake from nutrient-dense foods, in particular lean meats, and significantly lower percentage of energy intake from energy-dense nutrient-poor foods. The enhanced group reported lower daily energy intake at 12 months than at six months, while traditional group reported increased energy intake between six and 12 months.

The significant improvements in energy, sodium, and fruit intake achieved by the pooled intervention group compared with the control at six months align with results from other dietary interventions [11,57,58,59]. This suggests that adults with overweight or obesity can change these aspects of dietary intake in the short term when provided with evidenced-based advice. Reduced energy intake and increased fruit intake are associated with reduced risk of weight gain [60], and lower energy intake may assist in achieving clinically significant weight loss [61]. In turn, this may reduce multiple cardiometabolic risk factors including cholesterol, hypertension, and insulin sensitivity [62]. In addition, the reduced sodium intake to less than 2000 mg per day achieved by the pooled intervention group lowers cardiovascular disease risk [63], which is a leading cause of death [64].

This study observed that at 12 months, the pooled intervention group reported a non-statistically significant difference in energy intake relative to the control. This non-significant difference may be attributed to an increase in energy intake by the traditional group between six and 12 months. This indicates that on average, traditional group participants had difficulty in maintaining reduced energy intake beyond the active intervention period. Consequently, longer-term support such as face-to-face/telephone check-ins and social support may be needed, given that accountability has been reported as a strong facilitator of motivation and compliance with weight management behaviour change [65,66]. However, the enhanced group reported a positive shift in energy intake from nutrient-dense and energy-dense foods at 12 months. The two intervention groups did not differ in sleep quality at 12 months, but the enhanced group was the only group to receive the sleep intervention, and it also had the largest improvement in sleep quality from baseline to 12 months. Given that sleep duration is related to sleep quality [67] and short sleep duration influences dietary intake [27], the improved sleep quality of the enhanced group may have contributed to the reduced intake of energy-dense nutrient-poor foods. Although to date, research has focused on how sleep duration and not sleep quality influences dietary intake, and further research is needed to clarify this.

Overall, the results demonstrate that the sleep intervention did not produce significant improvements in indicators of sleep health relative to the traditional intervention. This may be because at baseline, both intervention groups reported sleep duration of ≈6.8 h per night, which was close to meeting the recommendation of 7–9 h per night for adults aged 18–64 years [68]. Relative to the much shorter sleep duration reported in meta-analyses that had an effect on dietary intake (≤5.5 h per night) [18,26,27], it may be that the participants’ sleep was not short enough and not improved enough during the intervention period to affect dietary behaviours (e.g., frequency of meals/snacks) and dietary intake. Furthermore, participants in the enhanced and traditional groups reported mean (SD) PSQI global scores of 7.3 (2.8) and 7.0 (3.1) at baseline, respectively. A PSQI global score of ≤5 indicates ‘good’ sleep quality, and a global score of >5 indicates ‘poor’ sleep quality, with a score of 21 representing the most severe sleep difficulty [53]. Therefore, it may be that the participants’ sleep quality, although classified as poor, was not impaired enough, and not improved enough in the enhanced intervention group to affect dietary intake. In this study, participants were recruited based on body weight status, rather than on physical activity, diet, and sleep behaviours. Future studies may benefit from specifically recruiting adults with overweight and obesity who are poor sleepers.

To the authors’ knowledge, this study is the only multiple-behaviour-change weight loss RCT to include a sleep health component and report dietary outcomes in the longer-term (i.e., 12 months). To our knowledge, existing RCTs have limited their investigation to the effect of extending sleep duration on a small number of dietary outcomes, and the studies were all ≤6 weeks in length [29,30,31,69]. This highlights the unique contribution of the current study, as it is the only published weight loss RCT to include a sleep health component that also measured a large number of dietary outcomes over a longer period. Additional longer-term (>6 months) studies are required to evaluate effects of improved sleep health on dietary intake, as sleep health is multi-dimensional, and changes in behaviour may take some time to become apparent [67]. In terms of overall dietary patterns, there was no significant difference observed between the groups in diet quality at six or 12 months, which is consistent with another sleep intervention [29]. The dietary intervention in this study primarily focused on increased intake of lower energy, nutrient-dense foods (e.g., fruits and vegetables) and reduced intake of energy-dense, nutrient-poor foods and drinks to achieve a reduction in daily energy intake, as reported by the enhanced group at 12 months. The dietary advice was less focused on increasing the food variety, and future dietary interventions are encouraged to focus on this to promote improvements in overall diet quality.

This study had a number of strengths including the RCT design, assessor blinding, validated outcome measures, comprehensive set of dietary outcomes assessed, and generalisability. This study also had some limitations. The study was powered to detect a difference between groups in the primary outcome of weight only, which increased the probability of a type 2 error. As multiple comparisons were performed, the chance of a type 1 error was also increased. The 95% confidence intervals are relatively wide for the between-group differences in total energy intake, sodium intake, and the percentage of energy intake from fruit at six months, and for a number of outcomes at 12 months, which indicates high variability in the between-group differences. As such, these results should be interpreted with some caution. A further limitation is the use of self-reported questionnaires and FFQs, which can result in misreporting, recall bias, measurement error, and vulnerability to social desirability bias [70]. However, the same tool was used to assess diet in all participants at all time points, although the AES could not detect changes in portion sizes, only consumption frequency.

## 5. Conclusions

This multi-component m-Health diet, physical activity, and sleep behaviour weight loss intervention significantly reduced energy intake and sodium intake, and it increased fruit intake in adults with overweight and obesity in the short term (six months). The enhanced group, who received an additional sleep health intervention, reported improved dietary intake relative to the traditional group in the longer term (12 months). Further adequately powered studies using longer-term sleep interventions are needed to examine whether improving sleep health is an effective strategy for improving dietary intake and weight management.

## Figures and Tables

**Table 1:** Baseline sociodemographic, health, and behavioural characteristics of participants by group.

Characteristics/Behaviours	Total	Control Group	Pooled Intervention Group	Traditional Group (*n* = 41)	Enhanced Group (*n* = 39)
M (SD)	(*n* = 116)	(*n* = 36)	(*n* = 80)		
Age (years)	44.5 (10.4)	40.5 (10.7)	46.3 (9.8)	45.4 (10.2)	47.2 (9.4)
Sex, n (%)					
Female	82 (70.7)	25 (69.4)	57 (71.2)	30 (73.2)	27 (69.2)
Weight (kg)	90.7 (14.3)	92.5 (16.1)	89.8 (13.4)	88.9 (13.8)	90.8 (13.1)
BMI (kg/m^2^)	31.7 (3.9)	31.9 (3.9)	31.7 (3.9)	31.4 (3.8)	31.9 (4.0)
Waist circumference (cm)	99.6 (11.0)	99.7 (11.7)	99.6 (10.8)	99.6 (9.0)	99.5 (12.5)
HbA1c (%)	5.4 (0.5)	5.3 (0.3)	5.5 (0.6)	5.5 (0.7)	5.5 (0.4)
Dietary intake					
Energy intake					
Total energy intake (kJ/d)	9683 (3146)	9153 (2810)	9922 (3274)	10,397 (2989)	9422 (3519)
Macronutrient intake					
Carbohydrate (%EI)	43.5 (6.8)	43.3 (8.3)	43.6 (6.0)	44.0 (5.3)	43.2 (6.7)
Fats (%EI)	33.3 (5.0)	33.8 (6.2)	33.0 (4.4)	33.7 (3.8)	32.2 (5.0)
Saturated fat (%EI)	14.0 (2.7)	14.4 (3.1)	13.8 (2.5)	14.3 (2.4)	13.2 (2.5)
Monounsaturated fat (%EI)	12.4 (2.2)	12.7 (2.8)	12.2 (1.92)	12.4 (1.6)	12.0 (2.2)
Polyunsaturated fat (%EI)	4.0 (1.0)	3.9 (1.0)	4.1 (1.0)	4.0 (0.9)	4.1 (1.2)
Protein (%EI)	18.0 (3.0)	18.0 (2.6)	18.0 (3.2)	17.9 (2.9)	18.2 (3.5)
Alcohol (%EI)	5.6 (7.1)	5.4 (7.3)	5.6 (7.0)	4.8 (6.5)	6.5 (7.4)
Micronutrient intake					
Sugars (g/d)	125.0 (57.7)	119.9 (56.4)	127.3 (58.5)	135.2 (54.0)	119.1 (62.6)
Fibre (g/d)	27.0 (8.9)	24.5 (8.4)	28.1 (9.0)	28.8 (8.1)	27.4 (9.9)
Sodium (mg/day)	2390.2 (858.1)	2310.0 (833.2)	2426.1 (871.9)	2598.6 (864.7)	2244.7 (852.9)
Nutrient-dense food intake					
Nutrient-dense foods (%EI)	58.3 (12.1)	56.1 (9.9)	59.3 (12.8)	59.5 (13.5)	59.1 (12.2)
Vegetables (%EI)	8.5 (3.9)	8.1 (3.3)	8.7 (4.2)	8.0 (4.1)	9.5 (4.1)
Vegetables (serves/day)	4.5 (1.7)	4.0 (1.3)	4.7 (1.7)	4.6 (1.6)	4.9 (1.9)
Fruits (%EI)	6.6 (4.2)	6.3 (4.2)	6.7 (4.1)	7.0 (4.2)	6.5 (4.2)
Fruits (serves/day)	1.8 (1.2)	1.8 (1.3)	1.8 (1.2)	2.0 (1.2)	1.7 (1.2)
Milk, yoghurt, cheese (%EI)	9.0 (5.3)	8.1 (4.8)	9.4 (5.5)	10.3 (5.5)	8.5 (5.4)
Breads, cereals, rice, pasta, noodles (%EI)	16.7 (5.8)	15.9 (6.6)	17.1 (5.4)	17.5 (5.7)	16.6 (5.2)
Lean meats, fish, poultry, eggs, nuts (%EI)	13.1 (6.2)	13.7 (6.1)	12.9 (6.2)	12.4 (5.9)	13.4 (6.8)
Protein alternatives, nuts, eggs, beans	4.4 (3.2)	4.4 (3.1)	4.4 (3.3)	4.2 (3.4)	4.5 (3.1)
Energy-dense food intake					
Energy-dense, nutrient-poor foods (%/day)	41.7 (12.1)	43.9 (9.9)	40.7 (12.8)	40.5 (13.5)	40.9 (12.2)
Fatty meats (%EI)	2.0 (1.6)	2.3 (2.0)	1.9 (1.4)	2.1 (1.6)	1.8 (1.3)
Fried/takeaway foods (%EI)	9.1 (5.6)	10.1 (5.5)	8.7 (5.7)	8.1 (4.9)	9.3 (6.5)
Confectionery (%EI)	6.8 (6.0)	6.5 (5.3)	6.9 (6.3)	8.1 (7.3)	5.6 (5.0)
Baked sweet products (%EI)	5.6 (4.2)	4.6 (3.4)	6.0 (4.4)	6.8 (4.2)	5.1 (4.5)
Packaged snacks (%EI)	3.0 (3.0)	3.4 (3.4)	2.8 (2.9)	2.8 (2.7)	2.9 (3.1)
Sweetened drinks (%EI)	3.0 (5.4)	4.7 (8.4)	2.2 (3.1)	1.8 (2.3)	2.5 (3.9)
Diet quality					
Total diet quality (ARFS 0–73)	35.4 (9.0)	33.3 (10.0)	36.3 (8.4)	36.0 (8.0)	36.5 (9.0)
Vegetables (ARFS 0–21)	14.2 (4.0)	13.4 (4.5)	14.7 (3.8)	14.4 (3.6)	15.0 (3.9)
Fruits (ARFS 0–12)	5.4 (2.8)	5.3 (3.2)	5.5 (2.7)	5.6 (2.3)	5.3 (3.1)
Protein foods—meat/flesh (ARFS 0-7)	2.7 (1.4)	2.5 (1.4)	2.7 (1.3)	2.6 (1.0)	2.9 (1.6)
Protein foods—meat/flesh alternatives (ARFS 0-6)	2.2 (1.2)	2.1 (1.2)	2.2 (1.3)	2.1 (1.2)	2.3 (1.3)
Grains, breads, and cereals (ARFS 0–13)	5.4 (1.9)	4.8 (1.9)	5.7 (1.8)	5.8 (1.7)	5.6 (1.8)
Dairy foods (ARFS 0–11)	3.9 (1.8)	3.6 (1.8)	4.0 (1.8)	4.0 (1.6)	4.0 (2.0)
Water (AFRS 0–1)	0.5 (0.5)	0.5 (0.5)	0.5 (0.5)	0.5 (0.5)	0.5 (0.5)
Extras (ARFS 0–2)	1.1 (0.7)	1.2 (0.7)	1.0 (0.8)	1.1 (0.8)	1.0 (0.7)
Physical activity					
MVPA (min/w)	312.8 (297.8)	238.1 (239.2)	346.4 (316.3)	351.0 (357.7)	341.5 (270.6)
Sleep health					
Sleep quality score (PSQI Global score)	7.0 (3.0)	6.7 (3.1)	7.1 (3.0)	7.0 (3.1)	7.3 (2.8)
Sleep duration (min/d)	406.0 (58.3)	406.7 (51.9)	405.8 (61.3)	408.3 (60.3)	403.1 (63.0)

**Abbreviations:** %EI, percentage of energy intake; ARFS, Australian recommended food score; d, day; g, grams; kJ, kilojoules; mg, milligrams; MVPA, moderate-to-vigorous intensity physical activity; PSQI, Pittsburgh Sleep Quality Index; w, week.

**Table 2:** Between-group differences in dietary intake, physical activity, and sleep health at six months (*n* = 81).

Intake/Behaviour	Control Group (*n* = 21)	Pooled Intervention Group (*n* = 60)	Between-Group Difference (95% CI)	*p*-Value	Effect Size (Cohen’s *d*)	Traditional Intervention Group (*n* = 32)	Enhanced Intervention Group (*n* =28) M (SE)	Between-Group Difference (95% CI)	*p*-Value	Effect Size (Cohen’s *d*)
	M (SE)	M (SE)				M (SE)				
Energy intake										
Total energy intake (kJ/d)	9198 (399)	8187 (235)	−1011 (−1922, −101)	0.029	0.55	8276 (316)	8318 (339)	42 (−895, 979)	0.93	0.02
Macronutrient intake										
Carbohydrate (%EI)	41.6 (1.3)	42.0 (0.8)	0.4 (−2.5, 3.4)	0.771	0.07	42.6 (1.0)	41.9 (1.1)	−0.7 (−3.6, 2.1)	0.617	0.12
Total fats (%EI)	33.6 (0.9)	32.7 (0.6)	−0.9 (−3.1, 1.2)	0.381	0.21	32.6 (0.7)	33.2 (0.8)	0.6 (−1.5, 2.6)	0.596	0.14
Saturated fat (%EI)	13.5 (0.5)	13.2 (0.3)	−0.3 (−1.4, 0.7)	0.555	0.15	13.2 (0.3)	13.4 (0.4)	0.1 (−0.9, 1.1)	0.782	0.05
Monounsaturated fat (%EI)	12.6 (0.4)	12.3 (0.2)	−0.3 (−1.3, 0.6)	0.523	0.15	12.2 (0.3)	12.6 (0.4)	0.4 (−0.6, 1.4)	0.433	0.21
Polyunsaturated fat (%EI)	4.0 (0.2)	4.2 (0.1)	0.2 (−0.2, 0.7)	0.378	0.25	4.2 (0.1)	4.3 (0.2)	0.03 (−0.4, 0.5)	0.894	0.04
Protein (%EI)	19.2 (0.7)	19.8 (0.4)	0.6 (−1.0, 2.2)	0.476	0.19	20.2 (0.6)	19.2 (0.6)	1.0 (−2.7, 0.7)	0.252	0.29
Alcohol (%EI)	5.6 (0.8)	5.6 (0.5)	0.05 (−1.8, 1.9)	0.961	0.01	4.6 (0.7)	5.6 (0.7)	1.0 (−1.0, 3.1)	0.305	0.26
Micronutrient intake										
Sugars (g/d)	113.6 (8.2)	103.2 (4.8)	−10.4 (−29.1, 8.2)	0.273	0.28	105.5 (5.5)	102.6 (5.9)	−2.8(−19.0, 13.4)	0.734	0.09
Fibre (g/d)	27.4 (1.2)	26.1 (0.7)	−1.3 (−4.0, 1.4)	0.332	0.24	27.6 (1.0)	25.9 (1.1)	−1.7 (−4.7, 1.2)	0.253	0.29
Sodium (mg/d)	2246.6 (122.1)	1933.4 (72.1)	−313.2 (−591.3, −35.0)	0.027	0.56	1983.8 (91.8)	1912.4 (98.6)	−71.4 (−344.3, 201.4)	0.608	0.13
Nutrient-dense food intake										
Nutrient-dense foods (%EI)	63.8 (2.1)	66.8 (1.2)	2.9 (−1.8, 7.7)	0.222	0.31	67.9 (1.8)	66.6 (2.0)	−1.2 (−6.6, 4.1)	0.654	0.12
Vegetables (%EI)	8.7 (0.8)	9.4 (0.5)	0.6 (−1.1, 2.4)	0.482	0.17	9.3 (0.7)	9.5 (0.8)	0.2 (−1.9, 2.2)	0.862	0.05
Vegetables (serves/d)	4.8 (0.3)	4.6 (0.2)	−0.2 (−1.0, 0.6)	0.703	0.13	4.9 (0.3)	4.5 (0.3)	−0.4 (−1.2, 0.5)	0.414	0.19
Fruits (%EI)	7.1 (0.9)	9.2 (0.5)	2.1 (0.1, 4.1)	0.04	0.53	9.9 (0.8)	8.3 (0.8)	−1.6 (−3.8, 0.6)	0.158	0.38
Fruits (serves/d)	1.8 (0.2)	2.2 (0.1)	0.3 (−0.2, 0.8)	0.233	0.25	2.4 (0.2)	1.8 (0.2)	−0.6 (−1.1, 0.02)	0.058	0.52
Milk, yoghurt, cheese (%EI)	10.5 (1.2)	10.0 (0.7)	−0.5 (−3.2, 2.1)	0.695	0.09	10.4 (0.7)	10.0 (0.8)	−0.4 (−2.6, 1.8)	0.727	0.09
Breads, cereals, rice, pasta, noodles (%EI)	17.0 (1.4)	17.3 (0.8)	0.3 (−2.9, 3.4)	0.862	0.05	16.9 (1.1)	18.7 (1.2)	1.8 (−1.5, 5.0)	0.287	0.27
Lean meats, fish, poultry, eggs, nuts (%EI)	14.9 (1.4)	15.6 (0.8)	0.7 (−2.5, 3.8)	0.674	0.11	15.7 (1.1)	14.6 (1.2)	−1.1 (−4.4, 2.1)	0.488	0.18
Protein alternatives, nuts, eggs, beans	5.2 (0.6)	5.6 (0.4)	0.4 (−1.0, 1.8)	0.581	0.14	5.5 (0.5)	5.8 (0.5)	0.3 (−1.0, 1.7)	0.618	0.11
Energy-dense food intake										
Energy-dense, nutrient-poor foods (%EI)	36.2 (2.1)	33.2 (1.2)	−2.9 (−7.7,1.8)	0.222	0.31	32.1 (1.8)	33.3 (2.0)	1.2 (−4.1, 6.6)	0.654	0.12
Fatty meats (%EI)	1.9 (0.3)	1.5 (0.2)	−0.3 (−0.9, 0.3)	0.31	0.25	1.4 (0.2)	1.4 (0.2)	−0.03 (−0.5, 0.4)	0.887	0.04
Fried/takeaway foods (%EI)	9.0 (0.8)	7.8 (0.5)	−1.2 (−3.0, 0.7)	0.214	0.34	8.4 (0.7)	7.2 (0.7)	−1.2 (−3.3, 0.8)	0.23	0.31
Confectionery (%EI)	4.4 (0.9)	5.0 (0.5)	0.6 (−1.4, 2.7)	0.537	0.15	4.1 (0.8)	6.3 (0.9)	2.2 (−0.2, 4.6)	0.076	0.47
Baked sweet products (%EI)	4.6 (0.7)	3.8 (0.4)	−0.8 (−2.3, 0.8)	0.338	0.25	4.5 (0.5)	3.6 (0.6)	−0.9 (−2.5, 0.7)	0.263	0.29
Packaged snacks (%EI)	2.4 (0.3)	1.7 (0.2)	−0.7 (−1.5, 0.02)	0.055	0.44	1.8 (0.2)	1.6 (0.3)	−0.2 (−0.9, 0.5)	0.563	0.13
Sweetened drinks (%EI)	2.0 (0.5)	1.1 (0.3)	−0.9 (−2.0, 0.2)	0.125	0.38	1.2 (0.3)	0.7 (0.3)	−0.5 (−1.2, 0.3)	0.214	0.32
Diet quality										
Total diet quality (ARFS 0-73)	36.2 (1.2)	36.7 (0.7)	0.5 (−2.1, 3.2)	0.687	0.09	37.6 (1.0)	37.2 (1.0)	−0.3 (−3.1, 2.4)	0.807	0.06
Vegetables (ARFS 0-21)	13.9 (0.5)	14.5 (0.3)	0.7 (−0.6, 1.9)	0.281	0.3	14.9 (0.5)	14.4 (0.5)	−0.5 (−1.8, 0.9)	0.483	0.18
Fruits (ARFS 0-12)	6.2 (0.5)	5.9 (0.3)	−0.3 (−1.5, 0.8)	0.551	0.13	6.3 (0.4)	5.6 (0.4)	−0.8 (−1.9, 0.4)	0.194	0.35
Protein foods—meat/flesh (ARFS 0-7)	2.5 (0.2)	3.0 (0.1)	0.5 (−0.1, 1.1)	0.08	0.42	3.0 (0.2)	2.9 (0.2)	−0.04 (−0.6, 0.5)	0.89	0.03
Protein foods—meat/flesh alternatives (ARFS 0-6)	2.1 (0.2)	2.4 (0.1)	0.3 (−0.2, 0.8)	0.221	0.38	2.3 (0.2)	2.5 (0.2)	0.2 (−0.3, 0.7)	0.346	0.26
Grains, breads, and cereals (ARFS 0–13)	5.5 (0.4)	5.5 (0.2)	−0.05 (−1.0, 0.9)	0.909	0.03	5.4 (0.3)	6.0 (0.3)	0.6 (−0.3, 1.5)	0.168	0.39
Dairy foods (ARFS 0–11)	3.9 (0.3)	3.7 (0.2)	−0.2 (−0.9, 0.6)	0.674	0.13	4.1 (0.2)	3.7 (0.3)	−0.3 (−1.0, 0.4)	0.37	0.19
Water (AFRS 0–1)	0.6 (0.1)	0.8 (0.04)	0.2 (−0.03, 0.3)	0.108	0.51	0.8 (0.1)	0.8 (0.1)	0.1 (−0.1, 0.3)	0.415	0.26
Extras (ARFS 0–2)	1.1 (0.1)	1.0 (0.1)	−0.1 (−0.4, 0.2)	0.388	0.13	0.7 (0.1)	1.1 (0.1)	0.4 (0.1, 0.7)	0.011	0.52
Physical activity										
MVPA (min/w)	393.1 (70.2)	455.8 (41.0)	62.7 (−98.2, 223.6)	0.445	0.19	535.5 (59.6)	402.6 (63.8)	−133.0 (−305.3, 39.4)	0.131	0.39
Sleep health										
Sleep quality score (PSQI Global score)	6.7 (0.7)	5.8 (0.4)	−0.8 (−2.4, 0.7)	0.292	0.25	6.1 (0.6)	5.7 (0.6)	−0.4 (−2.0, 1.3)	0.659	0.13
Sleep duration (min/d)	403.4 (11.9)	405.8 (7.0)	2.5 (−24.6, 29.5)	0.859	0.05	401.1 (10.0)	413.8 (10.7)	12.7 (−16.4, 41.8)	0.393	0.22

**Abbreviations:** %EI, percentage of energy intake; ARFS, Australian recommended food score; d, day; g, grams; kJ, kilojoules; m, mean; mg, milligrams; MVPA, moderate-to-vigorous intensity physical activity; PSQI, Pittsburgh Sleep Quality Index; SE, standard error; w, week. ***Note:*** Boldface indicates statistical significance (*p* < 0.05).

**Table 3:** Between-group differences in dietary intake, physical activity and sleep health at 12 months (*n* = 54).

Intake/Behaviour	Control Group (*n* = 17) M (SE)	Pooled Intervention Group (*n* = 37) M (SE)	Between-Group Difference (95% CI)	*p*-Value	Effect Size (Cohen’s *d*)	Traditional Intervention Group (*n* = 23) M (SE)	Enhanced Intervention Group (*n* = 14) M (SE)	Between-Group Difference (95% CI)	*p*-Value	Effect Size (Cohen’s *d*)
Energy intake										
Total energy intake (kJ/d)	9125 (450)	8211 (372)	−913 (−2033, 207)	0.11	0.47	8673 (490)	7949 (654)	−724 (−2389, 940)	0.394	0.29
Macronutrient intake										
Carbohydrate (%EI)	42.9 (1.9)	42.0 (1.0)	−0.8 (−5.2, 3.6)	0.715	0.11	43.6 (0.9)	40.5 (2.1)	−3.1 (−7.7, 1.5)	0.189	0.46
Total fats (%EI)	32.2 (1.1)	32.1 (0.7)	−0.1 (−2.7, 2.5)	0.931	0.02	31.6 (0.8)	33.3 (1.6)	1.7 (−1.8, 5.2)	0.341	0.32
Saturated fat (%EI)	13.3 (0.5)	12.9 (0.4)	−0.4 (−1.6, 0.9)	0.583	0.2	13.3 (0.5)	12.6 (0.6)	−0.7 (−2.3, 0.9)	0.41	0.3
Monounsaturated fat (%EI)	12.2 (0.5)	12.0 (0.3)	−0.1 (−1.0, 1.3)	0.811	0.05	11.5 (0.3)	12.8 (0.7)	1.3 (−0.3, 2.8)	0.107	0.55
Polyunsaturated fat (%EI)	3.6 (0.2)	4.1 (0.1)	0.4 (−0.1, 0.9)	0.119	0.39	3.9 (0.1)	4.4 (0.3)	0.5 (−1.2, 0.1)	0.103	0.56
Protein (%EI)	19.5 (1.0)	19.9 (0.5)	0.3 (−2.0, 2.6)	0.777	0.07	18.9 (0.5)	21.3 (1.1)	2.4 (0.1, 4.6)	**0.04**	0.74
Alcohol (%EI)	5.0 (1.0)	5.8 (0.5)	0.7 (1.4, 2.9)	0.508	0.19	5.7 (0.6)	4.6 (0.7)	−1.2 (−2.8, 0.5)	0.16	0.51
Micronutrient intake										
Sugars (g/d)	119.2 (13.1)	107.3 (5.8)	−11.9 (−40.0, 16.2)	0.407	0.24	114.6 (6.9)	99.5 (10.3)	−15.1 (−39.9, 9.6)	0.232	0.41
Fibre (g/d)	27.7 (1.4)	26.7 (1.1)	−1.0 (−4.5, 2.6)	0.586	0.16	27.4 (1.4)	27.8 (2.3)	0.3 (−5.3, 6.0)	0.908	0.04
Sodium (mg/d)	2265.3 (146.6)	1939.1 (93.0)	−326.2 (−662.6, 10.2)	0.057	0.56	2077.5 (130.2)	1814.7 (138.3)	−262.8 (−639.8, 114.1)	0.172	0.46
Nutrient-dense food intake										
Nutrient-dense foods (%EI)	62.9 (2.3)	66.0 (1.6)	3.1 (−2.5, 8.8)	0.281	0.31	63.9 (1.8)	71.3 (2.4)	7.4 (1.3, 13.5)	**0.017**	0.81
Vegetables (%EI)	9.6 (0.6)	10.1 (0.7)	0.4 (−1.3, 2.1)	0.619	0.13	9.4 (0.7)	11.1 (1.4)	1.7 (−1.6, 5.1)	0.312	0.34
Vegetables (serves/d)	5.0 (0.4)	4.9 (0.3)	−0.1 (−1.0, 0.7)	0.729	0.07	4.9 (0.4)	5.2 (0.4)	0.3 (−0.7, 1.4)	0.52	0.2
Fruits (%EI)	6.9 (0.8)	8.5 (0.6)	1.6 (−0.3, 3.5)	0.093	0.47	9.4 (0.8)	6.8 (1.0)	−2.6 (−5.3, 0.2)	0.066	0.63
Fruits (serves/d)	1.9 (0.2)	2.1 (0.1)	0.1 (−0.4, 0.7)	0.62	0.1	2.4 (0.2)	1.5 (0.2)	−0.8 (−1.5, −0.2)	**0.011**	0.9
Milk, yoghurt, cheese (%EI)	9.6 (1.2)	9.3 (1.1)	−0.3 (−3.4, 2.7)	0.824	0.06	10.2 (1.5)	8.9 (1.6)	−1.3 (−6.1, 3.5)	0.59	0.18
Breads, cereals, rice, pasta, noodles (%EI)	16.4 (1.4)	15.9 (1.0)	0.5 (−4.0, 2.9)	0.769	0.08	16.2 (1.2)	17.2 (2.1)	1.0 (−4.0, 6.0)	0.695	0.14
Lean meats, fish, poultry, eggs, nuts (%EI)	15.9 (2.0)	15.9 (1.0)	0.04 (−4.6, 4.5)	0.985	0.01	13.7 (0.9)	18.1 (1.9)	4.5 (0.4, 8.5)	**0.029**	0.76
Protein alternatives, nuts, eggs, beans	3.9 (0.5)	5.1 (0.6)	1.3 (−0.3, 2.9)	0.121	0.48	4.5 (0.5)	6.3 (1.4)	1.8 (−1.1, 4.7)	0.216	0.41
Energy-dense food intake										
Energy-dense, nutrient-poor foods (%EI)	37.1 (2.3)	34.0 (1.6)	−3.1 (−8.8, 2.5)	0.281	0.31	36.1 (1.8)	28.7 (2.4)	−7.4 (−13.5, −1.3)	**0.017**	0.81
Fatty meats (%EI)	1.5 (0.2)	1.6 (0.3)	0.05 (−0.8, 0.7)	0.883	0.04	1.8 (0.4)	1.1 (0.2)	−0.6 (−1.6, 0.3)	0.205	0.41
Fried/takeaway foods (%EI)	9.6 (0.9)	7.1 (0.6)	−2.4 (−4.7, −0.2)	0.034	0.64	8.4 (0.7)	4.8 (1.2)	−3.6 (−6.5, −0.7)	**0.015**	0.81
Confectionery (%EI)	4.8 (0.6)	4.5 (0.4)	−0.3 (−1.8, 1.2)	0.688	0.13	4.7 (0.6)	4.2 (0.7)	−0.4 (−2.2, 1.4)	0.64	0.15
Baked sweet products (%EI)	4.1 (0.5)	4.4 (0.4)	−0.3 (−1.1, 1.6)	0.696	0.13	5.5 (0.6)	3.5 (0.6)	−2.0 (−3.6, −0.4)	**0.014**	0.85
Packaged snacks (%EI)	2.8 (0.5)	1.7 (0.3)	−1.1 (−2.2, 0.1)	0.067	0.54	2.1 (0.4)	1.0 (0.3)	−1.1 (−2.2, −0.03)	**0.043**	0.75
Sweetened drinks (%EI)	2.9 (1.1)	1.8 (0,4)	−1.1 (−3.4, 1.1)	0.322	0.29	1.2 (0.4)	2.3 (0.8)	1.1 (−0.5, 2.7)	0.194	0.47
Diet quality										
Total diet quality (ARFS 0–73)	36.9 (1.5)	37.5 (1.2)	0.6 (−3.2, 4.4)	0.752	0.09	38.6 (0.8)	36.8 (2.7)	−1.8 (−7.4, 3.8)	0.532	0.22
Vegetables (ARFS 0–21)	15.3 (0.9)	15.7 (0.5)	0.4 (−1.6, 2.3)	0.725	0.12	15.6 (0.4)	16.0 (1.3)	0.4 (−2.3, 3.0)	0.779	0.1
Fruits (ARFS 0–12)	5.4 (0.5)	5.8 (0.4)	0.4 (−0.9, 1.7)	0.589	0.17	6.0 (0.4)	5.5 (1.0)	−0.5 (−2.6, 1.6)	0.645	0.15
Protein foods—meat/flesh (ARFS 0–7)	2.8 (0.2)	3.0 (0.1)	0.2 (−0.4, 0.7)	0.529	0.2	3.0 (0.2)	2.8 (0.2)	−0.2 (−0.8, 0.5)	0.632	0.23
Protein foods—meat/flesh alternatives (ARFS 0–6)	1.9 (0.2)	2.4 (0.2)	0.5 (−0.1, 1.1)	0.082	0.49	2.4 (0.2)	2.3 (0.4)	−0.1 (−1.0, 0.8)	0.811	0.07
Grains, breads, and cereals (ARFS 0–13)	5.7 (0.5)	5.3 (0.3)	−0.4 (−1.4, 0.7)	0.516	0.23	5.7 (0.3)	5.0 (0.4)	−0.7 (−1.8, 0.4)	0.229	0.4
Dairy foods (ARFS 0–11)	3.9 (0.4)	3.8 (0.2)	−0.1 (−0.9, 0.8)	0.852	0.07	4.5 (0.3)	3.2 (0.3)	−1.2 (−2.2, −0.3)	**0.011**	0.81
Water (AFRS 0–1)	0.7 (0.1)	0.7 (0.1)	0.03 (−0.2, 0.2)	0.805	0.09	0.6 (0.1)	0.8 (0.1)	0.2 (0.01, 0.4)	**0.037**	0.68
Extras (ARFS 0–2)	1.0 (0.1)	0.8 (0.1)	−0.2 (−0.5, 0.1)	0.241	0.29	0.8 (0.1)	0.9 (0.2)	0.1 (−0.4, 0.6)	0.614	0.17
Physical activity, M (SD)	395.1 (117.4)	433.8 (53.2)	38.7 (−218.5, 296.0)	0.768	0.09	525.2 (76.9)	358.3 (70.2)	−166.9 (−370.2, 36.4)	0.108	0.55
MVPA (min/w)										
Sleep health, M (SD)										
Sleep quality score (PSQI Global score)	5.8 (0.6)	5.1 (0.5)	−0.7 (−2.3, 0.9)	0.423	0.26	5.1 (0.7)	5.1 (0.6)	−0.02 (−1.8, 1.7)	0.977	0.01
Sleep duration (min/d)	420.8 (12.7)	422.2 (9.2)	1.4 (−30.2, 33.0)	0.931	0.03	425.5 (13.8)	422.9 (9.0)	−2.6 (−35.9, 30.6)	0.877	0.05

**Abbreviations:** %EI, percentage of energy intake; ARFS, Australian recommended food score; d, day; g, grams; kJ, kilojoules; m, mean; mg, milligrams; MVPA, moderate-to-vigorous intensity physical activity; PSQI, Pittsburgh Sleep Quality Index; SE, standard error; w, week. ***Note:*** Boldface indicates statistical significance (*p* < 0.05).

## Data Availability

Study data are available from the corresponding author (M.J.D.) upon request.

## Supplementary Materials

The following are available online at https://www.mdpi.com/article/10.3390/nu13072468/s1, Figure S1: CONSORT diagram describing study design and flow of participants.
