# Supplementary material for: Efficacy of a Multi-Component m-Health Diet, Physical Activity, and Sleep Intervention on Dietary Intake in Adults with Overweight and Obesity: A Randomised Controlled Trial
Source: Nutrients. 2021 Jul 19;13(7):2468. doi: 10.3390/nu13072468 (PMC8308779; doi:10.3390/nu13072468)
Supplement: Supplementary file 1 [file nutrients-13-02468-s001.zip › nutrients-1290119-supplementary.pdf]

Supplementary Material

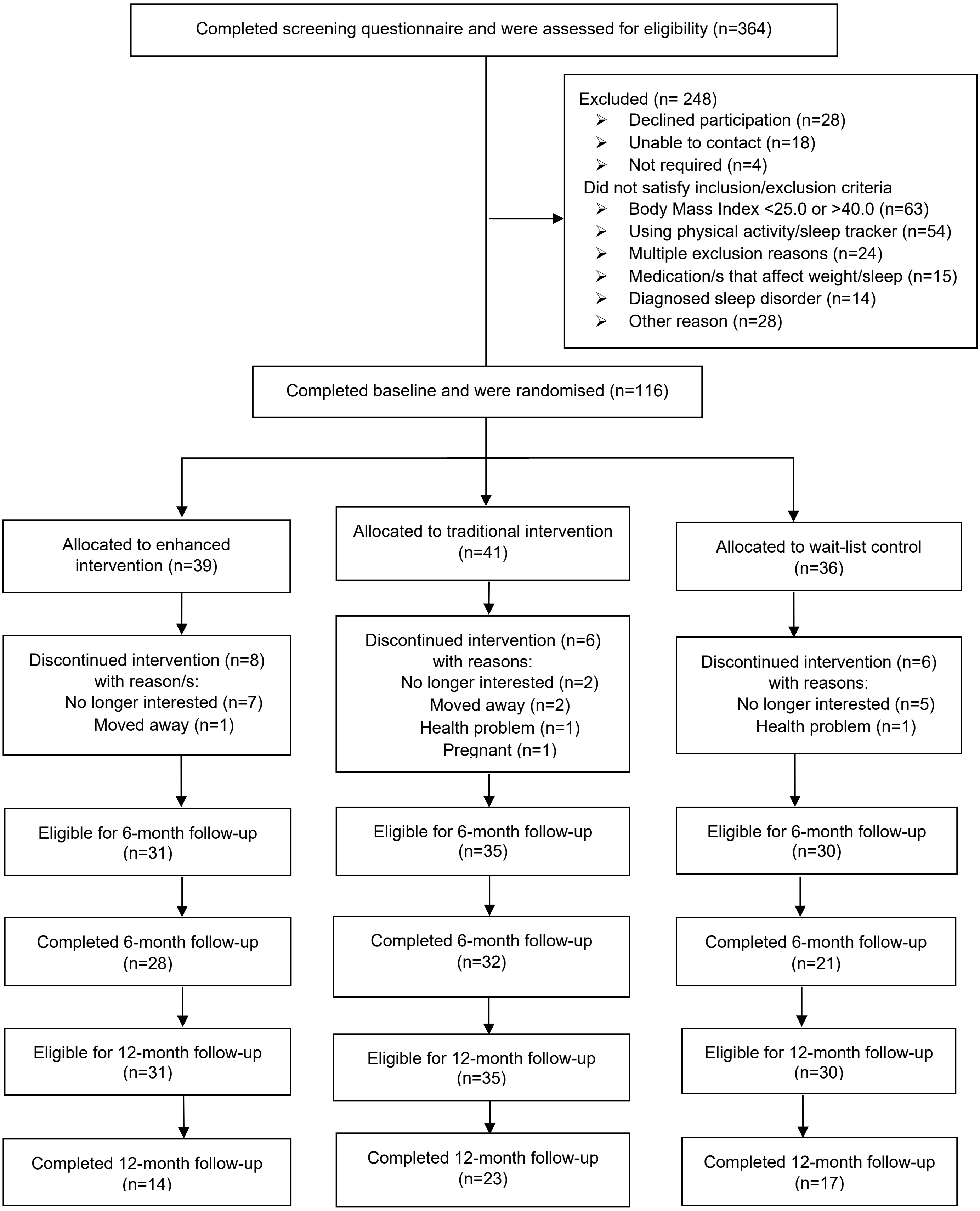

Figure S1. CONSORT diagram describing study design and flow of participants
